# Comparison between the Effects of Rocuronium, Vecuronium, and Cisatracurium Using Train-of-Four and Clinical Tests in Elderly Patients

**DOI:** 10.5812/aapm.8406

**Published:** 2013-03-26

**Authors:** Ozlem Sagir, Funda Yucesoy Noyan, Ahmet Koroglu, Muslum Cicek, Huseyin Ilksen Toprak

**Affiliations:** 1 Department of Anesthesiology and Reanimation, Faculty of Medicine, Balikesir University, Balikesir, Turkey; 2 Department of Anesthesiology and Reanimation, Yeşilyurt State hospital, Malatya, Turkey; 3 Department of Anesthesiology and Reanimation, Private Gazi Osmanpaşa Hospital, Istanbul, Turkey; 4 Department of Anesthesiology and Reanimation, Faculty of Medicine, Inonu University, Malatya, Turkey

**Keywords:** Neuromuscular Blockade, Aged, Rocuronium, Cisatracurium, Vecuronium Bromide

## Abstract

**Background:**

Postoperative residual blockade, longer duration of action for neuromuscular blockade, and slower recovery were relatively common in elderly patients.

**Objectives:**

We aimed to investigate the safety of train-of-four ratio and clinical tests in the assessment of patient recovery, and to determine the effects of the rocuronium, vecuronium, and cisatracurium on intubation, extubation and recovery times in elderly patients undergoing abdominal surgery.

**Patients and Methods:**

After obtaining institutional approval and informed consent, 60 patients over 60 years old and undergoing elective abdominal operations were included in this double-blind, randomized clinical trial. Following a standard anesthesia induction, 0.6mg kg-1 rocuronium, 0.1mg kg-1 vecuronium, and 0.1mg kg-1 cisatracurium were administered to the patients in Group R, Group V, and Group C, respectively. Train-of-four (TOF) ratios were recorded at 10-minute intervals during and after the operation. Modified Aldrete Score (MAS) and clinical tests were recorded in the recovery room at 10-minute intervals. In addition, intubation and extubation times, duration of recovery room stay, and any complications were recorded.

**Results:**

Intubation time was found to be shorter in Group R than that in Groups V and C (P ˂ 0.001). Times to positive visual disturbances and grip strength tests were shorter in Group C than that in Group V (P = 0.016 and P = 0.011, respectively). In Group R and group C, time to TOF ≥ 0.9 was significantly longer than all positive clinical test times except grip strength (P < 0.05).

**Conclusions:**

We hold the opinion that cisatracurium is safer in elderly patients compared to other drugs. We also concluded that the usage of TOF ratio together with clinical tests is suitable for assessment of neuromuscular recovery in these patients.

## 1. Background

In previous studies, clinically significant postoperative residual blockade, longer duration of action for neuromuscular blockade, and slower recovery were reported with intermediate-acting neuromuscular blocking drugs in elderly patients (1-4). Postoperative residual curarisation (PORC) may increase postoperative mortality and morbidity, and the incidence of respiratory side effects due to decreasing chemoreceptor sensitivity to hypoxia is a potentially lethal complication of anesthesia (4-7). Arterial oxygen tension is reduced with age as a ventilatory response to hypercapnia and hypoxia. This in turn leads to an increased risk of respiratory complications in elderly patients especially after abdominal surgery (3, 8, 9). Residual weakness of the jaw and tongue may cause difficulty in clearing secretions, and increase the risk of aspiration (4). Overall, there is an apparent confusion among clinicians around the best confirming method of recovery from neuromuscular blockade (4, 10, 11). In daily clinical practice, the most used neuromuscular monitoring methods are TOF stimulation and clinical tests (12, 13). There have been conflicting reports in the literature about evaluation of residual block using TOF. Some reports stated that even a TOF ratio ≥ 0.9 was not an accurate representation of neuromuscular status for recovery of patient from anesthesia (10, 12, 14). Others suggested that TOF ratio ≥ 0.9 would reflect acceptable neuromuscular recovery and decrease the risk of residual paralysis, postoperative atelectasis, and pneumonia following surgery (13, 15-19). Although there are studies on neuromuscular blocking drugs in the elderly, there is no controversial study using these three agents combining with TOF and clinical tests to evaluate neuromuscular recovery in the elderly patients undergoing abdominal surgery (1, 9, 20, 21).

## 2. Objectives

In our study, we aimed to investigate the reliability of TOF and clinical tests on recovery, and to determine the effects of rocuronium, vecuronium, and cisatracurium on intubation, extubation, and recovery times in elderly patients undergoing abdominal surgery.

## 3. Patients and Methods

After obtaining institutional approval and informed consent, we studied 60 patients with ASA physical status II-III, who were over 60 years old and underwent elective intra-abdominal operation. Estimated duration of surgery with general anesthesia was at least 180 minutes. The experimental design was a prospective, randomized, and double-blind study. Exclusion criteria were neuromuscular diseases, weight deviation more than 20% from ideal body weight, preoperative medication within last 72 hour that might interfere with neuromuscular transmission, severe liver and kidney diseases, electrolyte or acid-base disorders, alteration of plasma protein fraction, and endocrine disease with effects on metabolic rate. Patients were allocated using random number tables to receive rocuronium (Group R, n = 20), vecuronium (Group V, n = 20), and cisatracurium (Group C, n = 20) (Figure 1). Patients taken into the operation room without premedication were monitored by electrocardiography (ECG), pulse oxymeter, and noninvasive blood pressure. Mean arterial blood pressure (MAP), heart rate (HR), and peripheral oxygen saturation (SpO2) values were recorded before the operation. After an intravenous cannula was placed in the antecubital vein in the elbow that was not intended for neuromuscular monitoring, we started an intravenous infusion of isotonic saline 0.9%. Before the induction of anesthesia and with patient in the sitting position, a lumbar epidural catheter was inserted in the lumbar epidural space at L3-4 or L4-5 level using an 18-gauge Tuohy needle (B. Braun, Melsungen AG, Melsungen, Germany) with a loss of resistance technique under sterile conditions. Neuromuscular function was monitored assessing the contraction of the adductor pollicis muscle by accelerography (TOF-GUARD®, Organon-Teknika, Turnhout, Belgium). One arm was fixed and paediatric ECG surface electrodes were placed over the ulnar nerve at the wrist after cleansing the skin. The probe was positioned on the distal ventral part of the thumb; the other fingertips were tightly fixed with tape. Free movement of the thumb was ensured. Skin temperature over the adductor pollicis muscle was maintained > 32˚C by wrapping the arm in cotton wool. The ulnar nerve was stimulated with TOF stimulation (4 pulses 0.2 ms in duration, at a frequency of 2 Hz, 2 s in duration) at 10-s intervals. The current intensity was 40 mA. In anesthesia induction, propofol 2 mg/kg (Propofol1%®, Fresenius Kabi, Austria), fentanyl 2 µg kg-1 (Fentanyl Citrate®, Abbot, USA), lidocaine 1mg kg-1 (Aritmal 2%®, Biosel İlaç Sanayi, Istanbul, Turkey) were used in all cases. After anesthesia induction and loss of consciousness and prior to neuromuscular drug administration, accelerometer was calibrated using 0.2 ms supra-maximal square wave impulses at 0.1 Hz. After loss of eyelash reflex, rocuronium 0.6 mg kg-1, vecuronium 0.1 mg kg-1, and cisatracurium 0.1 mg kg-1 were given. The drugs were applied by anesthetist 1 who was not involved in any of the assessments. Tracheal intubation was performed when no response to nerve stimulation was seen at TOF- GUARD display. Anesthesia was maintained 4-6 % desflurane and 50% N2O in oxygen. Subsequent neuromuscular blockade was maintained to a depth of no responses to TOF using repeated doses of rocuronium 0.1 mg kg-1 and 0.02 mg kg-1 cisatracurium and vecuronium. During the operation if MAP or HR decreased by > 25% over pre-induction values, IV ephedrine 5.0 mg or atropine 0.5 mg was given. Ventilation was assisted to maintain end-tidal CO2 levels within a range of 35-40 mmHg. Body temperature was monitored by placing an esophageal probe and was kept over 35˚C using a warming blanket. Neuromuscular blockade was antagonized at the end of surgery with 50µg kg-1 neostigmine and 20µg kg-1 atropine, when two responses to TOF stimulation and twitch height recovery above 25% were present. The trachea was extubated when four responses to TOF stimulation were present, the patient regained consciousness, and the anesthetist judged that the neuromuscular function recovered adequately for upper airway protection and spontaneous ventilation. Postoperative analgesia provided at a rate of 6mL h-1 continuous infusion containing 0.25% bupivacaine with 1µgm L-1 fentanyl was given after surgery. ECG, SpO2, and TOF monitoring was continued after the operation in the recovery room. TOF ratios were recorded at 10 min intervals by a member of the team (anesthetist 2) who was blinded to group allocation. Modified Aldrete Score (MAS) and clinical tests were recorded at 10 min intervals by anesthetist 2. The following clinical tests were performed: 1. Visual disturbances (muddy vision, inadequacy of extra-ocular muscle function); 2. Facial weakness (difficulty to laugh, facial numbness); 3. Weakness of oral and pharyngeal muscles (swallowing); 4. Head lift for 5 seconds without assistance; 5. Sustained leg lift for 5 seconds; 6. Grip strength for 5 seconds; and 7. Retained tongue depressor. The time between the reversal of neuromuscular blocking drugs and the detection of positive clinical tests was recorded as positive clinical test times. The duration passed for the MAS score greater than 8, was recorded as time to MAS > 8. Mean arterial blood pressure, HR, SpO2 were recorded by anesthetist 1 at 10 min intervals from anesthesia induction until the end of operation and in recovery room. Intubation time was defined as the time from starting of neuromuscular blocking drugs injection to no response to TOF stimulation. Duration of TOF ≥ 0.9 was defined as the time interval between the extubation and a TOF ratio of 0.9. Duration of anesthesia was defined as the time between anesthesia induction and tracheal extubation, and extubation time was accepted as the time between the end of operation and tracheal extubation. Duration of surgery was defined as the time from anesthesia induction to closure of the skin. Intubation and extubation times, duration of anesthesia and surgery, and recovery room stay were recorded by a member of the team (anesthetist 3) who was blinded to group allocation. In the recovery room, all patients received 2 L dk-1 by a nasal cannula. SPO2 < 90% was accepted as hypoxia and 6 L dk-1 oxygen was given via a face mask by ensuring airway. In addition, complications encountered during and after operation were recorded. When MAS > 8 and TOF ≥ 0.9 were present and all clinical tests were positive, patients were transferred to the ward. Statistical analyses were performed using SPSS® 12.0 (SPSS inc. Chicago, IL, USA). Power analysis was performed using NCSS 2000 and PASS 2000 (NCSS Statistical & Power Analysis Software, Kaysville, UT). After preliminary study was performed with seven patients in each group, the power of the study was calculated based on the intubation and extubation times. Sample sizes of 17 subjects were obtained from three groups whose means were compared. The total sample of 51 subjects achieved 83% power to detect differences among the means versus the alternative of equal means using an F test with a 0,05 significance level. The size of variation in the means was represented by their standard deviation. Intergroup statistical analyses were performed using Kruskal-Wallis, Bonferroni correction, and Mann-Whitney U tests. Values within group assessed using Wilcoxon test and nonparametric-data were analyzed using X2-test. Results were presented as median (minimum-maximum). P < 0.05 was considered statistically significant for all tests.

## 4. Results

The patients characteristics, extubation time, duration of anesthesia and surgery, and recovery room stay did not differ between groups. Intubation time was significantly shorter in Group R than that in Groups V and C (P < 0.001) (Table 1). Times to positive grip strength and visual disturbances were shorter in Group C than that in Group V (P = 0.011, P = 0.016, respectively) (Table 2). In Group R, time to TOF ≥ 0.9 was significantly longer than times to MAS > 8 and all positive clinical test times except grip strength test (P < 0.05). Times to MAS > 8 was significantly longer than weakness of oral and pharyngeal muscles tests and shorter than times to visual disturbances, sustained leg lift, and grip strength tests (P < 0.05) (Table 3). In Group V, time to TOF ≥ 0.9 was significantly shorter than times to positive grip strength test and longer than head lift, and weakness of oral and pharyngeal muscles tests. Times to MAS > 8 was significantly longer than weakness of oral and pharyngeal muscles tests and shorter than visual disturbances, sustained leg lift, grip strength, and retained tongue depressor tests (P < 0.05) (Table 3). In Group C, time to TOF ≥ 0.9 was significantly longer than times to MAS > 8 and times to positivity in all clinical tests except grip strength test (P < 0.05). Times to MAS > 8 was significantly longer than weakness of oral and pharyngeal muscles tests and shorter than time to positivity of all clinical tests except head lift test (P < 0.05) (Table 3). MAP and HR were within normal limits and no complications were observed in either group during or after the operation.

**Table 1:** The Patients’ Characteristics, Intubation and Extubation Time, Duration of Anesthesia, Surgery and Recovery Room Stay

	Group R, Median (Minimum-Maximum), (n=20)	Group V, Median (Minimum-Maximum), (n=20)	Group C Median (Minimum Maximum), (n=20)	P value
**Age, y**	67.5 (60-80)	65 (60-80)	67 (60-80)	0.561
**Weight, Kg**	65 (60-90)	60 (40-100)	69 (50-100)	0.163
**Sex, male/female**	12/8	6/14	10/10	0.153
**Duration of anesthesia, min**	239.5 (163-328)	226(162-376)	209.5 (166-367)	0.514
**Duration of surgery, min**	222.5 (157-318)	214 (160-352)	191 (155-349)	0.624
**Intubation time, min**	3.2 (1.2-4.2)	5.4 (4.1-6.5)	5 (4.4-7.5)	< 0.001
R-V				< 0.001
R-C				< 0.001
V-C				0.195
**Extubation time, min**	4 (2-9)	4.5 (2-12)	4 (2-8)	0.432
**Duration of recovery room stay, min**	90 (40-120)	90 (40-120)	70 (30-110)	0.123

**Table 2:** Times to MAS > 8, TOF ≥ 0.9 and Positive Clinical Tests and Comparisons among the Groups a

	Group R, Median (Minimum-Maximum), (n=20)	Group V, Median (Minimum-Maximum), (n=20)	Group C, Median (Minimum-Maximum), (n=20)	P value
**MAS > 8**	50 (30-90)	50 (30-90)	40 (30-60)	0.770
**TOF ≥ 0.9**	90 (20-120)	60 (20-120)	65 (20-120)	0.116
**Visual disturbances **	60 (40-100)	65 (40-90)	50 (40-80)	0.044
R-V				0.353
R-C				0.089
V-C				0.016
**Facial weakness **	50 (30-90)	60 (30-90)	50 (30-70)	0.283
**Weakness of oral and pharengeal muscles **	30 (30-60)	30 (30-60)	30 (30-40)	0.083
**Head lift **	60 (30-110)	50 (30-110)	50 (30-70)	0.248
**Sustained leg lift **	60 (30-110)	60 (30-100)	50 (30-80)	0.058
**Grip strength **	70 (40-120)	80 (40-120)	65 (30-100)	0.049
R-V				0.390
R-C				0.188
V-C				0.011
**Retained tongue depressor **	60 (30-90)	70 (40-90)	55 (30-80)	0.075

Abbreviations: MAS, Modified Alderete Score; TOF, Train of four

^a^Bonferroni correction value (α*) = 0.016

**Table 3:** Times to MAS > 8, TOF ≥ 0.9 and Positive Clinical Tests and Comparisons within Groups

	MAS > 8	TOF ≥ 0.9	VD	FW	WOP	HL	SLL	GS	RTD	P value
**Group R**	50 (30-90)	90 (20-120)	60 (40-100)	50 (30-90)	30 (30-60)	60 (30-110)	60 (30-110)	70 (40-120)	60 (30-90)	
MAS-TOF										< 0.001
MAS-VD										0.03
MAS-WOP										< 0.001
MAS-GS										< 0.001
MAS-SLL										0.026
TOF-VD										0.002
TOF-FW										< 0.001
TOF-WOP										< 0.001
TOF-SLL										0.001
TOF-HL										< 0.001
TOF-RTD										< 0.001
**Group V**	50 (30-90)	60 (20-120)	65 (40-90)	60 (30-90)	30 (30-60)	50 (30-110)	60 (30-100)	80 (40-120)	70 (40-90)	
MAS-VD										0.001
MAS-WOP										0.001
MAS-GS										< 0.001
MAS-SLL										0.003
MAS-RTD										0.001
TOF-WOP										0.002
TOF-GS										0.017
TOF-HL										0.027
**Group C**	40 (30-60)	65 (20-120)	50 (40-80)	50 (30-70)	30 (30-40)	50 (30-70)	50 (30-80)	65 (30-100)	55 (30-80)	
MAS-TOF										0.001
MAS-VD										0.001
MAS-FW										0.001
MAS-WOP										0.001
MAS-GS										< 0.001
MAS-SLL										0.017
MAS-RTD										0.001
TOF-VD										0.028
TOF-FW										0.004
TOF-WOP										< 0.001
TOF-HL										0.001
TOF-SLL										0.001
TOF-RTD										0.009

Abbreviations: FW, Facial weakness; GS, Grip strength; HL, Head lift; MAS, Modified Alderete Score; RTD, Retained tongue depressor SLL, Sustained leg lift; TOF, Train of four; VD, Visual disturbances; WOP, Weakness of oral and pharengeal muscles

## 5. Discussion

Our findings indicated that although intubation time was faster with rocuronium, times to positive of grip strength and visual disturbances test were shorter with cisatracurium. Neuromuscular monitoring was available in every operating room; routine use of a quantitative neuromuscular monitoring is made only by around 10% of anesthetists (3, 4). Clinical evaluation of recovery, which depends on the degree of consciousness and cooperation of the patient, is not often performed correctly, and it does not solely exclude clinically significant residual curarisation after general anesthesia (4, 13, 16). Because of these reasons, we used both TOF ratio and clinical tests to assess neuromuscular recovery in our study by applying rocuronium, vecuronium, and cisatracurium in clinically potency doses. It has been stated that the onset time of non-depolarizing neuromuscular blockers is delayed in the elderly because of slower biophase equilibration. However, there were conflicting reports of action onset of non-depolarizing neuromuscular blockers in the elderly (1, 3, 21, 22). The onset time of rocuronium, vecuronium, and cisatracurium in the elderly has been defined by several investigators as 168-270 s, 158-295 s, and 150-270 s, respectively (3, 21). Keles et al. suggested that the onset time was significantly shorter with vecuronium than that with cisatracurium (20). In a study by Ghodraty et al. it was concluded that magnesium was able to hasten the muscle-relaxing effect of cisatracurium (23). In our study, intubation time was faster with rocuronium, which correlated with previous studies in the literature. Matteo et al. claimed that time to 90% recovery was 74.4 min after rocuronium (21). Claudius et al. found that the time to reach TOF > 0.9 and extubation time after a single intubating dose of 0.6 mg kg-1 from rocuronium was 224 min (2). Baykara and colleagues found the time to first response and four responses to TOF with rocuronium in elderly patients were 60.8 min and 83 min, respectively (1). Arain and colleagues reported that the durations of action was significantly shorter with cisatracurium; according to their findings, the medians of action durations for vecuronium, rocuronium, and cisatracurium were 62.4 min, 63.1 min, 56.8 min, respectively (22). Keles et al. reported that the time to TOF ≥ 0.9 was 88 min and 96 min for cisatracurium and vecuronium, respectively (20). It was stated that the times to 25% recovery for rocuronium and vecuronium in the elderly were 54 min and 50 min, respectively (3). Baillard and colleagues claimed that recovery time after vecuronium was 131 min (24). Our findings were compatible with previous studies in that the time to TOF ≥ 0.9 was similar in all groups. Previous reports have shown a discordance between the results of clinical neuromuscular tests and a TOF ratio of 0.9 (10, 13, 15, 16). Debane et al. reported that 24 patients could not sustain a 5-second head lift and 16 patients could not retained tongue depressor while TOF ratio ≥ 0.9 was present (25). Using clinical tests of motor function such as eye opening, tongue protrusion, and ability to cough and swallow to detect partial paralysis proved to be less satisfactory, whereas ability to sustain head or leg lift (and strong hand grip) proved to be reasonably reliable indicators for recovery and correlated well with TOF responses (3, 26). Claudius et al. found that the time to positivity in the clinical tests after 0.6 mg/kg single intubating dose of rocuronium was 265 min (2). Our findings differed from those of Claudius et al, in that our times to positive clinical tests were shorter. Also, times to positive visual disturbances and grip strength tests were shorter with cisatracurium compared to vecuronium. Cisatracurium could be more suitable in the elderly because the time to positive some clinical tests were shorter. In this study, the times to MAS > 8, TOF ≥ 0.9, and positive all clinical tests were not compatible with each other. Therefore, we believe that the use of TOF ratios, clinical tests, and MAS alone are not reliable to assess neuromuscular recovery. However, future studies are required to evaluate the reliability of TOF, MAS, and clinical tests to prevent PORC in elderly patients. It has been reported that incidence of postoperative residual blockade with intermediate- acting neuromuscular blocking drugs was 3.5-64% in elderly patients (26, 27). The deterioration of renal and hepatic functions associated with aging affects the clearance and elimination of vecuronium. Therefore, it may have a significantly prolonged duration of action in elderly patients (3). However, it has been proposed that since cisatracurium besilate undergoes predominantly organ independent elimination, clinical duration of action does not alter significantly in the elderly (3, 28). A case of severe residual blockade after single intubating dose of rocuronium was reported by Claudius and colleagues (2). It was reported that rocuronium, vecuronium, and cisatracurium had no significant effects on the heart rate or blood pressure (3, 9, 20). However, Clendenen and colleagues stated that an anaphylactic reaction after cisatracurium occurred in one case at normal clinical dosage and that hemodynamic side effect might occur depending on dose-related histamine release (29). In our study, no postoperative residual blockade or any other complications were observed. Drugs used via lumbar epidural catheter for postoperative analgesia might affect some clinical tests. Limitation of our study was inability to evaluate this situation. Another limitation of our study was that we used TOF for assessing tracheal intubation and patient recovery times; however, there are more sensitive tests in neuromuscular monitoring such as single-twitch and double burst stimulations which could be implemented. In conclusion, intubation time was faster with rocuronium. Times to positive grip strength and visual disturbances tests were shorter in cisatracurium compared to vecuronium. Therefore, cisatracurium may be a more suitable neuromuscular blocker in the elderly patients. Also, combined use of TOF ratio, MAS, and all clinical tests could be safer to assess neuromuscular recovery.
